# Health and Disease Are Dynamic Complex-Adaptive States Implications for Practice and Research

**DOI:** 10.3389/fpsyt.2021.595124

**Published:** 2021-03-29

**Authors:** Joachim P. Sturmberg

**Affiliations:** ^1^Faculty of Health and Medicine, School of Medicine and Public Health, University of Newcastle, Callaghan, NSW, Australia; ^2^International Society for Systems and Complexity Sciences for Health, Waitsfield, VT, United States

**Keywords:** health, philosophy of medicine, somato-psycho-socio-semiotic model of health, physiology, psychoneuroimmunology, complex adaptive systems, non-linear dynamics, systems thinking

## Abstract

Interoception, the ability to convey one's overall physiological state, allows people to describe their health along an experiential continuum, from excellent, very good, good, fair to poor. Each health state reflects a distinct pattern of one's overall function. This assay provides a new frame of understanding health and disease as complex-adaptive system states of the person as-a-whole. It firstly describes how complex patterns can emerge from simple equations. It then discusses how clinical medicine in certain domains has started to explore the pattern characteristics resulting in the heterogeneity of disease, and how this better understanding has improved patient management. The experiential state of health can be surprising to the observer—some are in good health with disabling disease, others are in poor health without the evidence of any. The main part of the assay describes the underlying complexity principles that contribute to health, and synthesizes available evidence from various research perspectives to support the philosophic/theoretical proposition of the complex-adaptive nature of health. It shows how health states arise from complex-adaptive system dynamics amongst the variables of a hierarchically layered system comprising the domains of a person's macro-level external environment to his nano-level biological blueprint. The final part suggests that the frame of health as a *dynamic complex-adaptive state* defines a *new paradigm*, and outlines ways of translating these expanded understandings to clinical practice, future research, and health system design.

Every man has his particular way of being in good health.

-Emanuel Kant

## Introduction

Since time in memoriam has health been regarded as a multidimensional complex-adaptive state (1)—a state that arises from the many non-linear interactions between its macro to nano-level variables (2). People have always experienced their health—as much in the presence as absence of diseases—in some patterned way in the context of their societal and environmental settings (Table 1).

**Table 1:** The Patterned Understanding of Health.

	**Description**	**Patterns arise from**
Plato (3)	Health is an application to human nature in all its parts, operations, levels, and dimensions—the physical, psychological, social, and spiritual	Interactions amongst four domains of health
Husserl (4)	Health is a holistic ability to relate properly to and function well in the whole lifeworld in all its aspects, and disease a disturbance of this ability, on any of a variety of levels or in any of a variety of dimensions	Interactions amongst domains focused on the maintenance of a stable state
Illich (5)	Health is a positive state that dynamically spans across the stages of life - The ability to adapt to changing environments, to growing up and to aging, to healing when damaged, to suffering and to the peaceful expectation of death	Temporal dynamic interactions amongst variables of self and one's environment
Antonovsky (6)	The sense of coherence is a global orientation that expresses the extent to which one has a pervasive, enduring though dynamic feeling of confidence that one's internal and external environments are predictable	Adaptive interactions amongst variables that define one's internal and external environment
Ingstad (7)	Health depends on many interconnected aspects of life: belonging to one's local environment/land, the sense of freedom, cultural and spiritual belonging, and the sense of dignity and security	Adaptive interactions amongst variables that define one's internal and external environment, especially those of personal self/sense

However, the medical discourse for too long has single-mindedly focused on health and disease arising from linear processes rather than entertaining the idea that health and disease arise from systemic interactions amongst their constituent parts. Indeed, a system is not the sum of behaviors of its parts, rather it is the *product of the interactions amongst its parts* (2). Hence the complex dynamics amongst the—external and internal—parts of a person result in identifiable (and often well-recognized) patterns which are correlated to his/her future trajectories. In turn these patterns govern clinical care and should be the object of future research endeavors.

This paper approaches health and disease understandings as much from a philosophical/theoretical perspective supported by a synthesis of available scientific evidence from across the health sciences fields. The first part briefly introduces the sciences of *pattern formation* as a way to understand heterogeneous outcomes. It alludes to some of the findings of applying pattern understandings to differentiate distinctive patterns *within* a disease, and its implication for disease management and outcomes.

While patterns of disease precede the discussion of health patterns the paper's focus is on the *complex adaptive patterns* of *health experiences*. Interoception—the ability to sense the internal state of our bodily function—allows us to convey our ever changing *health experiences* (8). Viewed over time our health experiences lead to well-recognized health patterns. Understanding these health patterns has practical applications to the care of patients, research and health system organization.

## Pattern Formation—A Mathematical Explanation

**Figure 1:**
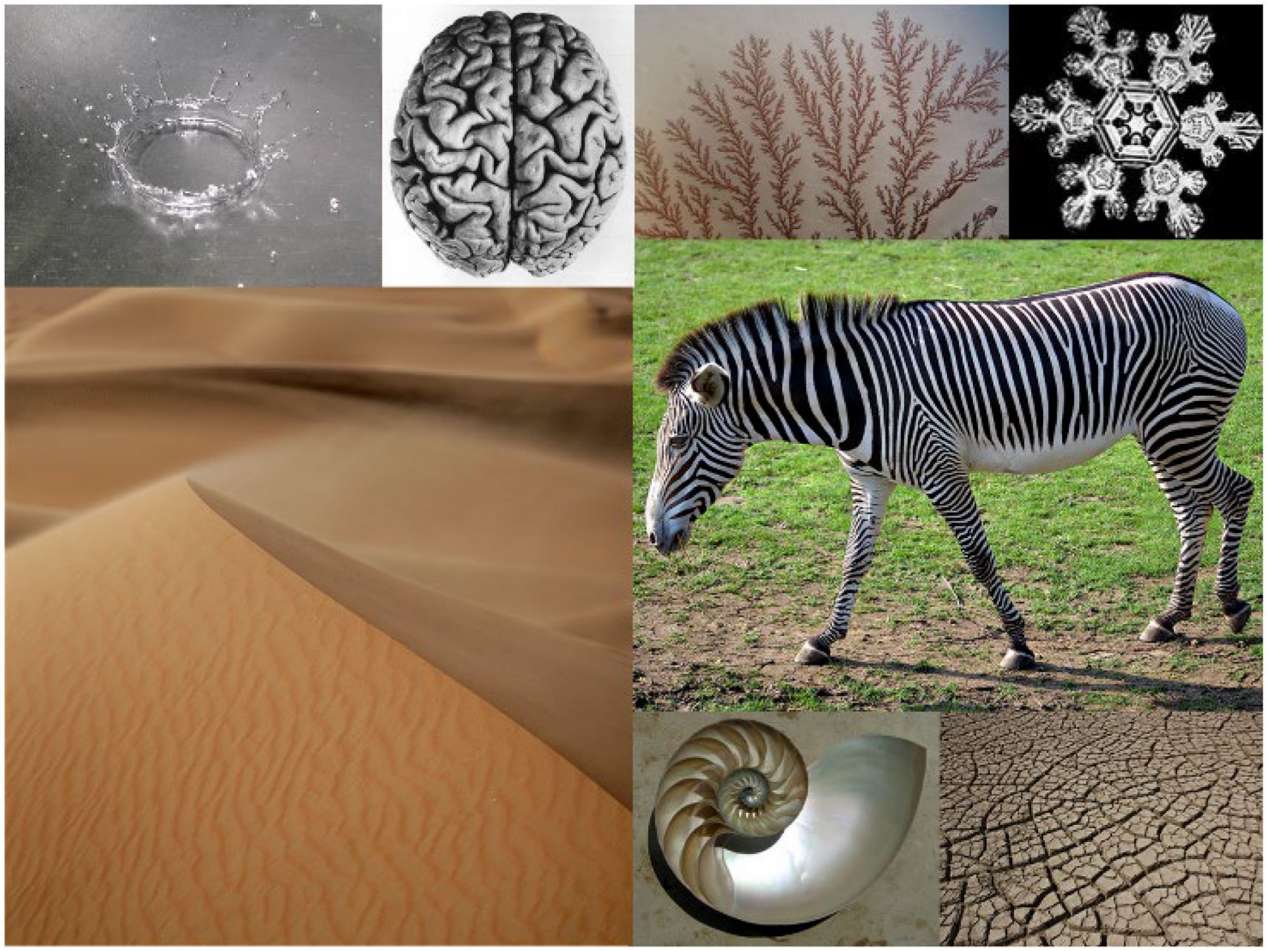
Patterns in Nature from the microscopic to the macroscopic scale (Images courtesy of Wikimedia, CC BY-SA 4.0).

Patterns describe similarities and differences in the world over time and space, and are the visible outcomes of emergent self-organization (9). Mathematically self-organization in complex systems is associated with *bifurcation*, i.e., a complex system reaches a state in space and time where multiple possible solutions are feasible which split the system into new stable states—a phaenomenon widely observed in nature (Figure 1).

**Figure 2:**
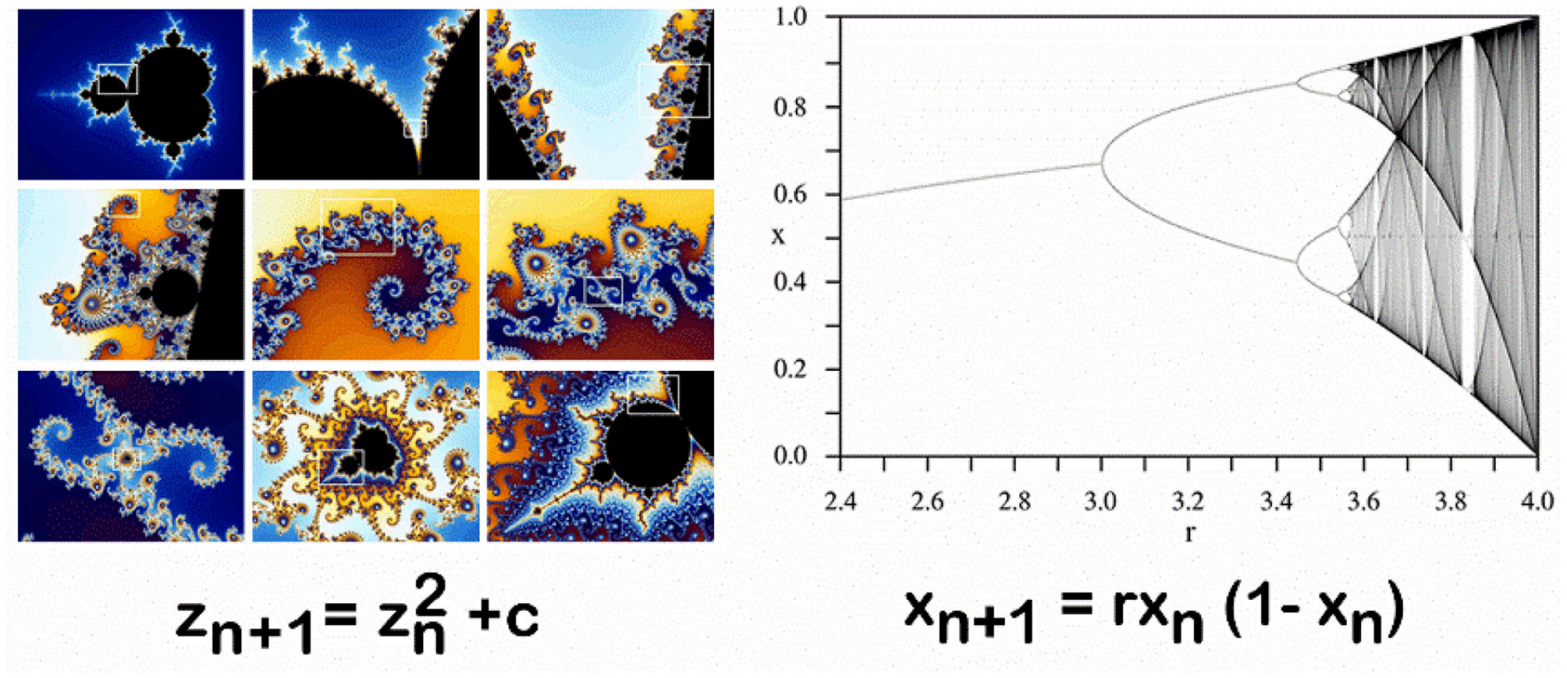
Simple Functions result in Complex Patterns (Images courtesy of Wikimedia, CC BY-SA 4.0).

Even complex patterns often arise from surprisingly simple mathematical functions—like $z_{n+1}=\;z_{n}^{2}\;+c$ the function of the Mandelbrot set or *x*_*n*+1_ = *rx*_*n*_ (1− *x*_*n*_) the function of a logistic map that results in a bifurcation diagram (Figure 2). The logistic map function shows how bifurcation results in both stable variability as well as chaos, and that chaos ultimately emerges again to stability (10, 11). Bifurcation is a common feature of dynamic systems, and, as Prigogine suggested, may well-provide “*the physio-chemical basis for understanding pathological behavior and disease”* (9, 12). In particular, bifurcation points can emerge from within a possible narrow range of parameter values of a system's agents (9), a point explored later in the paper.

## Disease Patterns—A Largely Descriptive Categorisation

Medical discourse frequently refers to patterns—at the global/policy levels as “life-course patterns of education/work environment/abuse/social class on health/morbidity,” at the community level as “disease patterns are changing in a region/state,” at the disease level as the “pattern of psoriasis,” the “patterns of Crohn's disease” or the “patterns of lung disease in high resolution CT scans,” and at the person level as “patterns of behaviors in eating/exercising/risk taking.” While these descriptive accounts tacitly embrace the heterogeneity of medical phaenomena, it has not inspired the exploration of the underlying principles of such pattern formation.

Clinicians only recently realized that their treatment approaches are suboptimal or fail for failing to consider heterogeneity amongst patients, morphological characteristics of a disease, and drivers of disease dynamics (13–18). Three examples illustrate how non-linear research approaches led to new insights. *Cluster analysis* has revealed that six variables (glutamate decarboxylase antibodies, age at diagnosis, BMI, HbA1c, β-cell function and insulin resistance) related to 5 distinct diabetes clusters with significantly different disease progression and diabetes complications (nephropathy, retinopathy) (13, 14). Four domains (motor, autonomic dysfunction, rapid eye movement behavior disorder, and cognitive dysfunction) distinguish three Parkinson Disease subtypes with distinct patterns of survival, falls, wheelchair use, onset of dementia and care placement patterns (15). At the microlevel cluster analysis has identified 8 intratumoural subtypes of glioblastoma multiforme based on the patterns of 9 immune markers (18).

Non-linear dynamic models like *Catastrophe* (from the French term meaning “jump”) *Models* help us to understand the sudden—often unexpected switch—between various stable states. In general terms *Catastrophe Theory* states that if we know the number of observed states we also know the number of different control parameters involved (note: a control parameter can be a combination of synergistic factors). The *Cusp Catastrophe Model* explains the sudden—discontinuous—change between TWO otherwise stable states of a phaenomenon, like experiencing good or poor health, being in a stable or unstable disease state (e.g., heart failure), or being able or unable to maintain a certain behavior (e.g., eating or drinking disorders). Each stable state contains many different sets of control parameter combinations, however, between these two stable states lies a “bifurcation point B” (the point where the variables of the three axes meet) that defines the origin of a highly unstable zone. Here small changes in either control parameter lead to an abrupt shift between the two stable states. Two examples illustrate these non-linear dynamics and their impact on a person's state of well-being: Sudden shifts in the recurrence of alcohol abuse is precipitated by the degree of “risk recurrence” (situational threats to self-efficacy, affective states, stressful life events, loss of social/family support, acute psychological distress) and the degree of “relapse predisposition” (family history of alcoholism, nature and severity of alcoholism, comorbid psychiatric and substance abuse diagnosis) (19), whereas the risk of committing suicide amongst HIV affected patients in China is precipitated by the degrees of “experience of stigma” and the degree of “social capital” (social support, social networks, trust in others) (20).

**Figure 3:**
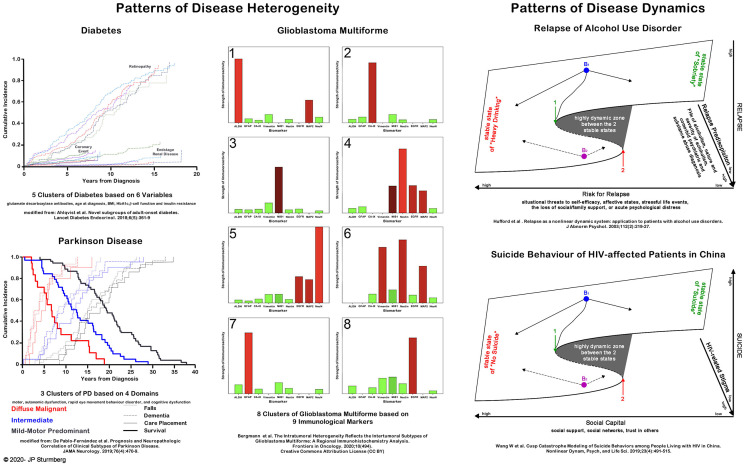
Application of disease pattern and dynamics understanding to clinical care. The left panel shows disease heterogeneity and its impacts at the macroscopic (diabetes and Parkinson Disease) and the molecular level (glioblastoma multiforme). The right panel demonstrates the discontinuous dynamics of disease behavior (alcohol use disorder and suicide risk). For more details on *cluster analysis* see Kohonen (21) and Amato et al. (22) and *cusp catastrophe models* see Thom (23) and Zeeman (24).

Understanding the properties behind the pattern formation of disease heterogeneity, and appreciating that small differences in a variable can result in very different disease trajectories is fundamental in our quest to find best possible treatment options as much for the “dis-eases” as the “diseases” of individual patients (Figure 3).

## Health Patterns—The Outcome of Complex-Adaptive Dynamics

A short detour into the theory of complex adaptive systems. Living systems are structurally bounded, thermodynamically open systems—while they constantly interact with their environment across their boundaries, boundaries also provide the space for their internal (physiological) function. An organism thus is structurally bounded/constraint by its physical embodiment, while simultaneously—at the macro-level—being part of its environment, and—at the micro-level—being the dynamic assembly of its constituent parts. This “multi-level” configuration provides the “necessary space” for the vital physiological functions that keep the organism alive (25). Of note, the function of an organism, while itself bounded, requires the function of its lower level units based on their own rules (25), an observation that has been generalized by Ellis as “top-down causation” in complex adaptive systems (26). Put differently, a complex adaptive system—like an organism—is the product of the interactions of its constituent parts, and its overall properties can neither be inferred from the parts nor are they present in the parts (2).

Back to health. As already alluded to, health is an *experiential state* of one's being as much in the presence as absence of disease (1), and it arises from our capacity for interoception as the means to convey our ever changing internal state of bodily function (8). As such *health*—from the old English word “hal”–is the *state of being whole*, and *dis-ease*—from the old French “des” and “ease”–describes the state of *loss of wholeness*. The now objectified use of “disease” relates to—mostly—visible pathologies (1, 27–29).

### The Dynamics of Health Experiences

If health is an experiential state arising from the dynamic multidimensional interactions between one's somatic, social, emotional, and cognitive domains, one's health can only be appreciated *as-a-whole*—not really a new insight:

**Table:** 

**Phaedrus:** Hippocrates the Ascelepiad says that the nature even of the body can only be understood as a whole.
**Socrates:** Yes, friend, and he is right – still we ought not to be content with the name of Hippocrates, but to examine and see whether his argument agrees with his conception of nature.
Phaedrus, 270

**Figure 4:**
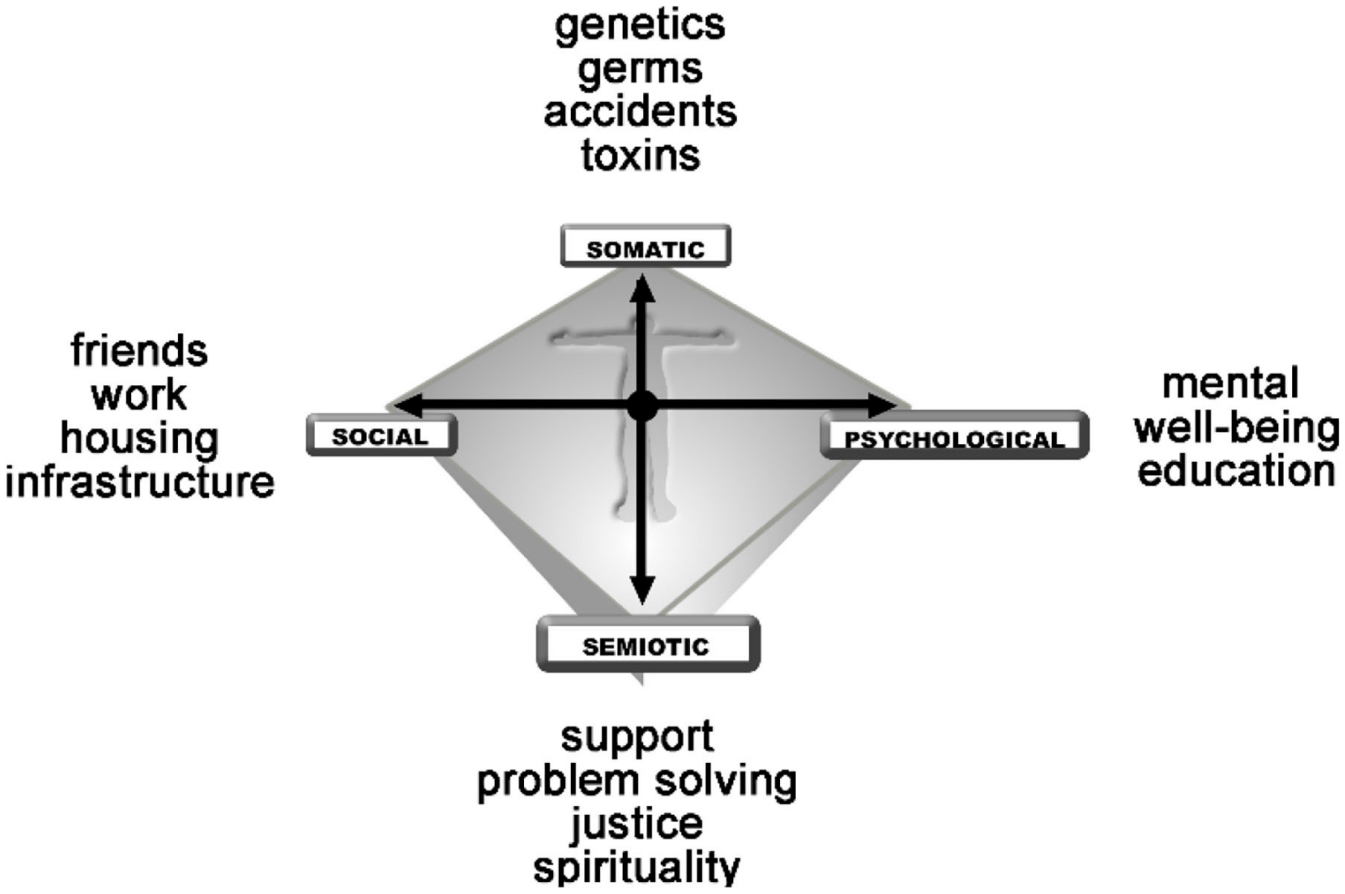
The SPSS-model of health defined as a balance between ones somatic (bodily), psychological (emotional), social and semiotic (sense-making) domains (32).

How then can we understand health as a dynamic state? Uexküll and Pauli (30) first described an integrated model of health that more accurately described the dynamics of the health experience constrained in a person's unique context. This model extended Engel's more limited biopsychosocial model (31). Sturmberg broadened these concepts and defined the SPSS (somato-psycho-socio-semiotic) model of health which emphasizes the dynamic interrelationships between these four key domains of human health experiences (1, 32) (Figure 4). This complex adaptive systems model takes account of the observational findings that some people have very significant burdens of (objective) disease but still experience *good health* whereas others without any obvious disease experience *poor health* (the dynamics of health and dis-ease fit a cusp catastrophe model).

**Figure 5:**
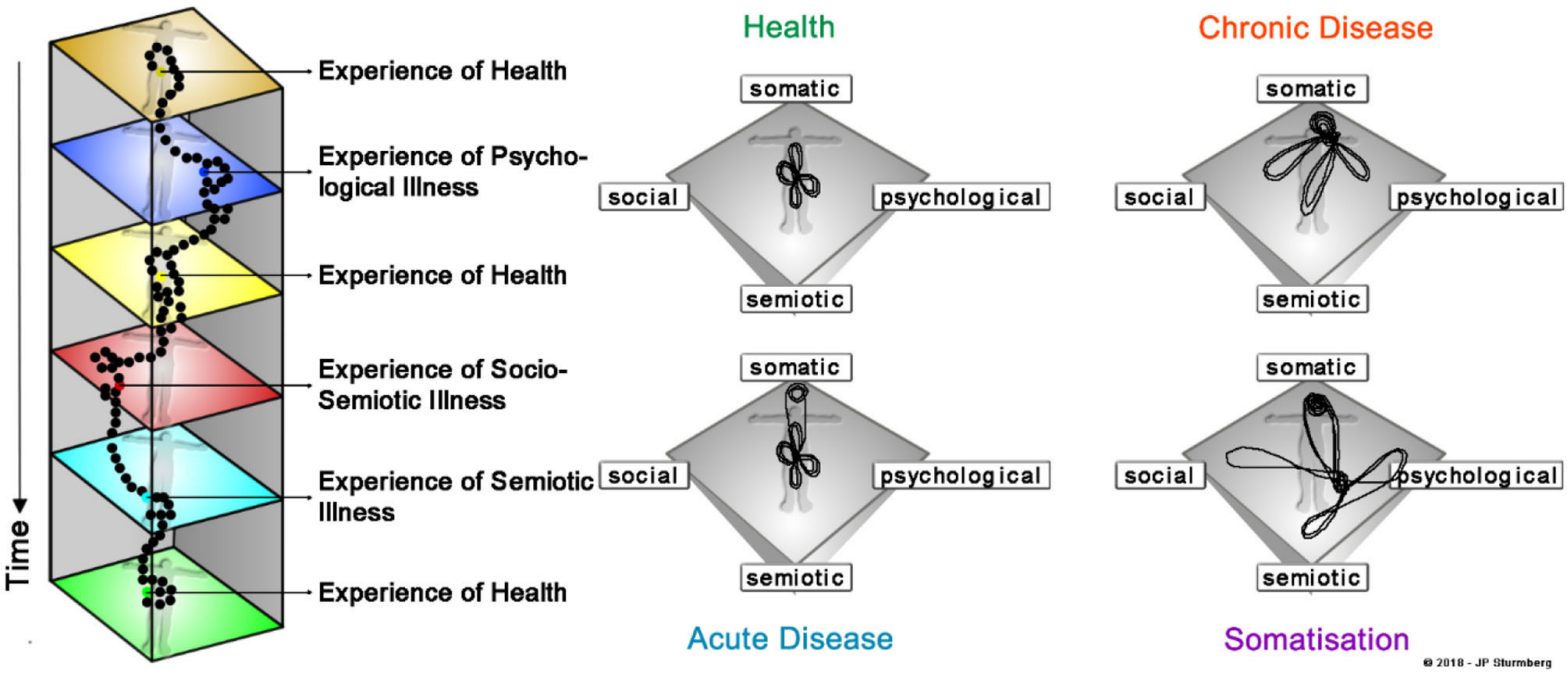
The dynamics of common health patterns. Our health experience results from the interactions between the four key domains of health—our physical, social, emotional and cognitive-sense making experiences. What impacts on our health experience varies—usually only slightly – from day to day (left panel). Collapsing the timeline into a “phase space” displays the dynamic patterns of well-known health states—*health* is characterized by a “balance between the four domains,” *acute disease* entails a sudden perturbation in the somatic domain which rapidly settles to restore the prior “balanced state” *chronic disease* is characterized by a shift of the balance to one domain associated with intermittent larger perturbations in any of the other domains (the example here represents typical somatic multimorbidity), and *somatisation* being characterized by an abrupt switch between somatic and emotional/cognitive-sense making states.

The dynamic interactions over time—plotted in a *phase space*—between the domains of health result in commonly observable *patterns* of health states—a *state of health* characterized by narrow dynamic fluctuations; a *state of acute self-limiting disease* that reverts back to a state of health; a *state of chronic disease* characterized by a shift into one domain and depending on circumstances associated with larger and longer shifts into condition-dependent other domains; and lastly a *state of psychosomatic illness* characterized by intermittent changes of health between domains (Figure 5). These states are *dynamic* and therefore *changeable* (in line with the principles of bifurcation and cusp catastrophe)—at times spontaneously, at others related to sudden changes in life circumstances, and at times due to the interventions of health professionals who share a person's health journey.

### Physiological Dynamics—Bounded Between the Environment and the Biological Blueprint

While the SPSS-model describes the outcomes of dynamic interactions amongst key health domains in terms of *phenotypes*, it does not in itself explain the underlying interactions amongst the various agents within and across its domains. This requires consideration of a range of different concepts, like Ashby's law of *requisite variety* (cybernetics), Rothman's notion of *multiple sufficient causes* of a condition (generally speaking) being a precursor leading to the understandings of *networked* interdependencies between system layers (network physiology), the inflammatory cascades as the principal regulator to maintain homeokinetic stability (psychoneuroimmunological), and Ellis's already mentioned notion of *top-down causation* in complex adaptive systems (philosophy of complex adaptive systems theory).

#### Ashby's Law of Requisite Variety

Ashby's law of requisite variety states that for a system to remain stable it needs to have a “reservoir of correcting responses” at least as great as it has “challenges”—failing this the system will become unstable or even fail (33). Complex biological systems are constantly challenged by internal and external perturbations. They have developed highly effective adaptive responses to such challenges, however, as complex biological systems age they lose their adaptive capacity, or put in Ashby's terms, they lose their requisite variety (34). The trajectory of aging, and with it the accumulation of “definable diseases,” is a stepwise process characterized by the loss of “optimal stability” at one point in time leading to an adapted “new state” of temporary—*homeokinetic*—*stability*, a process that repeats many times over the lifespan (35). The loss of homeokinetic stability, and with it the increase in disease development and ultimately frailty, is associated with another system characteristic—the increase in system entropy. All biological systems ultimately reach a level of entropy incompatible with life (36, 37).

#### Multiple Sufficient Causes

**Figure 6:**
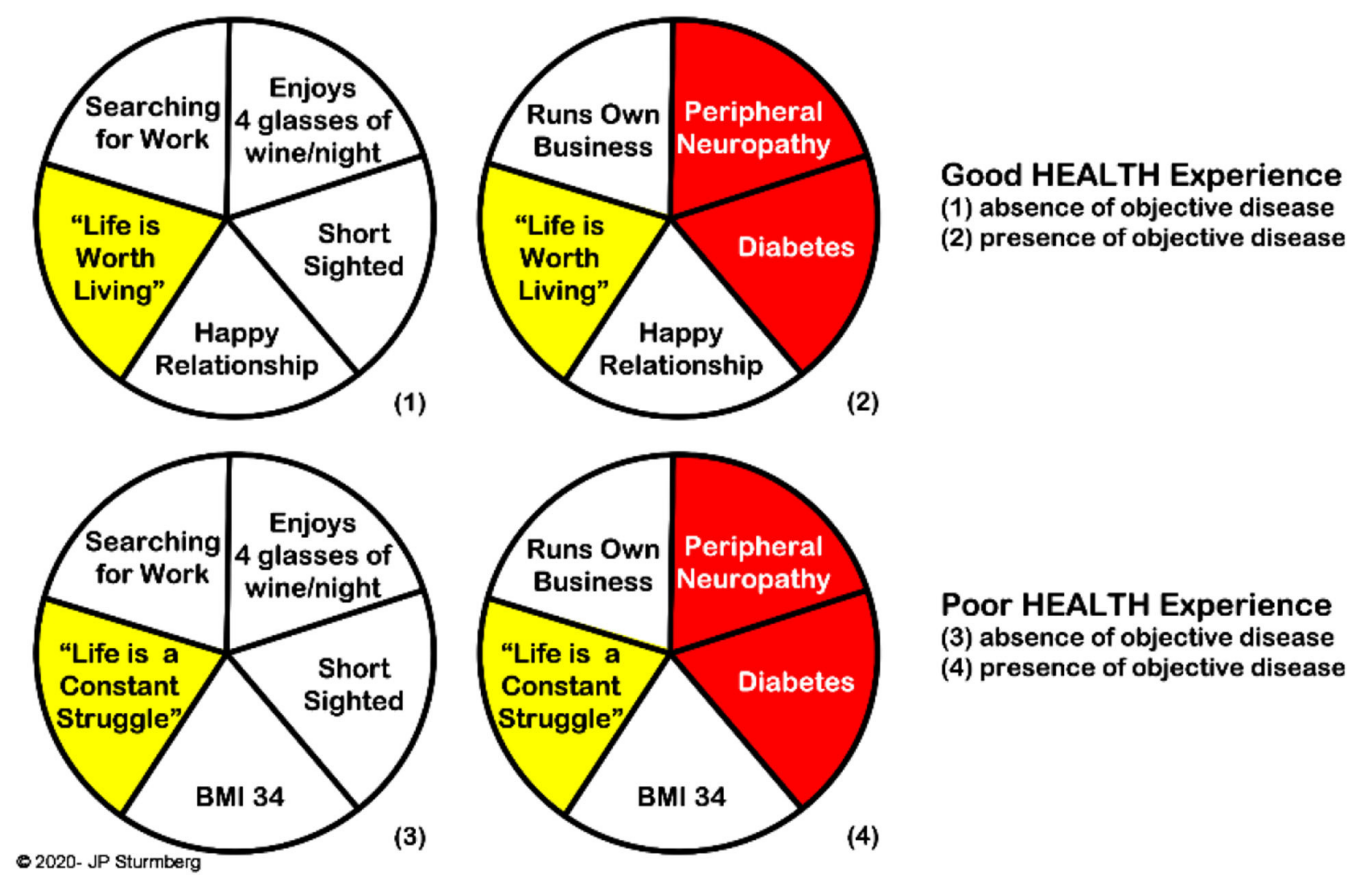
Rothman's model of necessary and sufficient causes of outcomes. “Appreciation of life” is the *necessary* cause to the experience of health regardless of other *sufficient* causes which may or may not include “objective disease.” The model arises from the mathematics of “set theory.”

In 1976 Rothman alluded to the heterogeneity of causes resulting in an *outcome*—a disease, a condition, or any other specific health outcome. He pointed out that a particular outcome can be caused by various *sets of sufficient* causes (referring to set theory). A cause that is present in all sets of sufficient causes is a *necessary* cause, and typically forms part of the “causative definition” for an outcome (38). The example in Figure 6 illustrates that “appreciation of life” is the *necessary* cause for a person's health experience. The presence of disease—diabetes and its complications—is only part of a set of *sufficient* causes to contribute to the outcome of *good* and *poor* “health experience.”

#### Network Physiology

**Figure 7:**
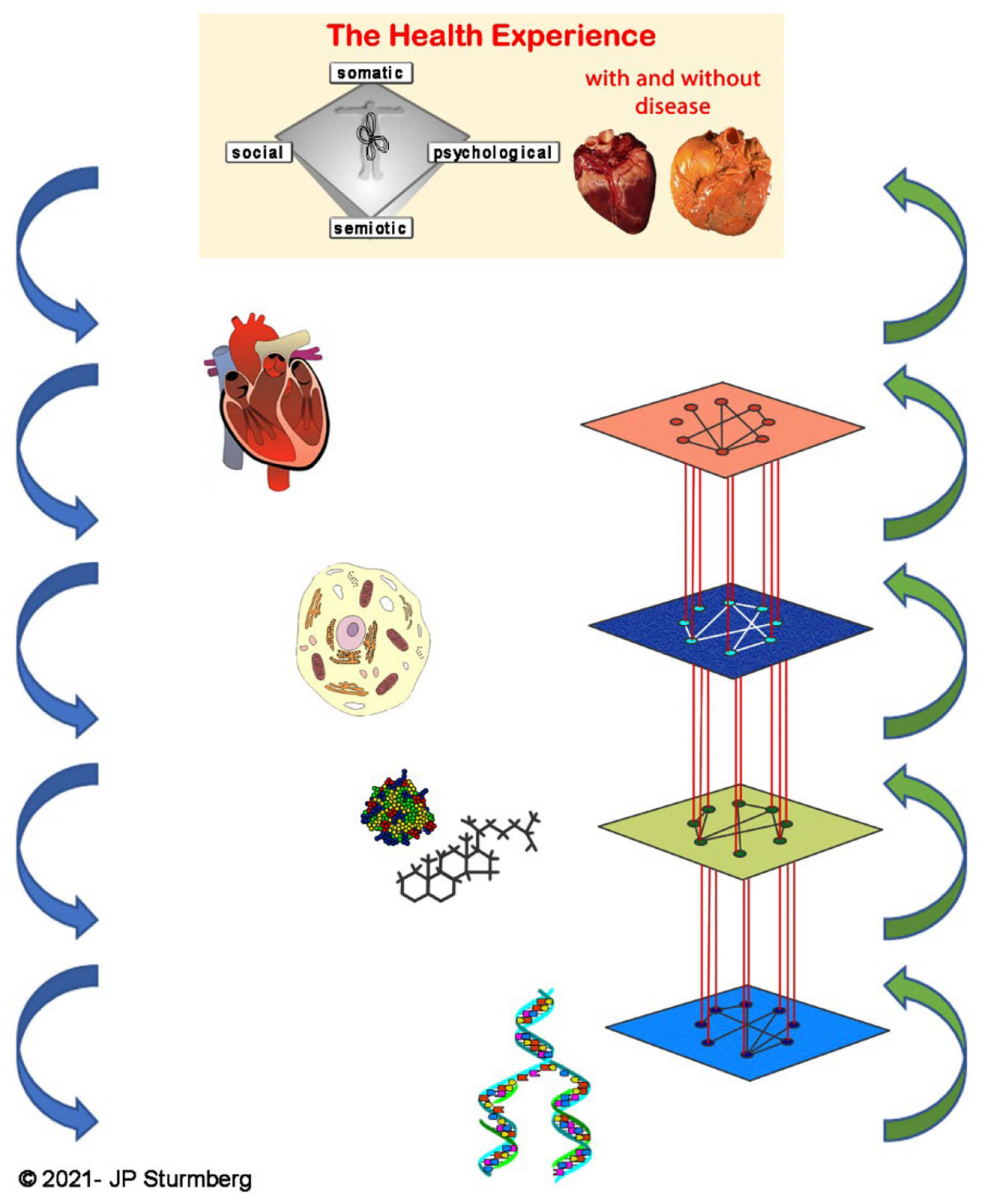
Network physiology. Network physiology describes the spatio-temporal system integration and their dynamics the various layers of a biological system—organ, cell, metabolome and genome.

Conceptually, network physiology is the “microscopic” as well as “dynamic” extension of the “macroscopic” perspective of Rothman's model of sufficient causes. Rather than simply describing the phenomenological characteristics associated with health and disease, network physiology aims to elucidate the *spatio-temporal system integration* and the *dynamics* between the organ, cell, metabolomic and genomic layers of biological systems (Figure 7) (39). Each layer has its own distinctive dynamics which influences the dynamic interactions across layers. Alteration in the interactions within a layer will not only change the dynamics of that layer but also those of interacting layers, and thereby shift the integrated function of the *system-as-a-whole* (39, 40). Physiological network properties have pragmatic clinical implications as exemplified in patients with liver failure (41) or severe critical illness (42)—the breakdown/loss of network interconnection between the layers of system organization is associated with poor outcomes.

#### Inflammatory Regulation

Inflammatory pathways *regulate* most of our internal body functions. The hypothalamic-pituitary-adrenal (HPA) axis controls cortisol, epinephrine, norepinephrine and acetylcholine levels which regulate immune cell function. Depending on their stimulation immune cells release pro- and antiinflammatory cytokines. Cytokines are as much implicated in gene as mood regulation besides of the local inflammatory responses. Dysregulation of these pathways [as well as some lipid based mediators (43)] leads to a prolonged or chronic low-grade inflammatory system state that initially leads to sub-optimal adaptive homeokinetic stability (44, 45), but over time damages tissues and organ systems to such a degree that recognizable disease emerges (46–49).

Of note, the so-called *illness behavior*—anorexia/cachexia, anhedonia, cognitive alterations, fatigue, depressed mood and pain—is triggered largely by peripheral proinflammatory cytokines causing neuroglial inflammation and the release of high brain-derived cytokine levels. In the resolution phase of an acute illness antiinflammatory cytokines reverse the brain inflammation and the person returns to his pre-illness state. However, the chronically elevated cytokine levels associated with chronic disease results in chronic neuroglial inflammatory activity resulting in the lower mood and higher fatigue levels of affected patients (50, 51).

**Figure 8:**
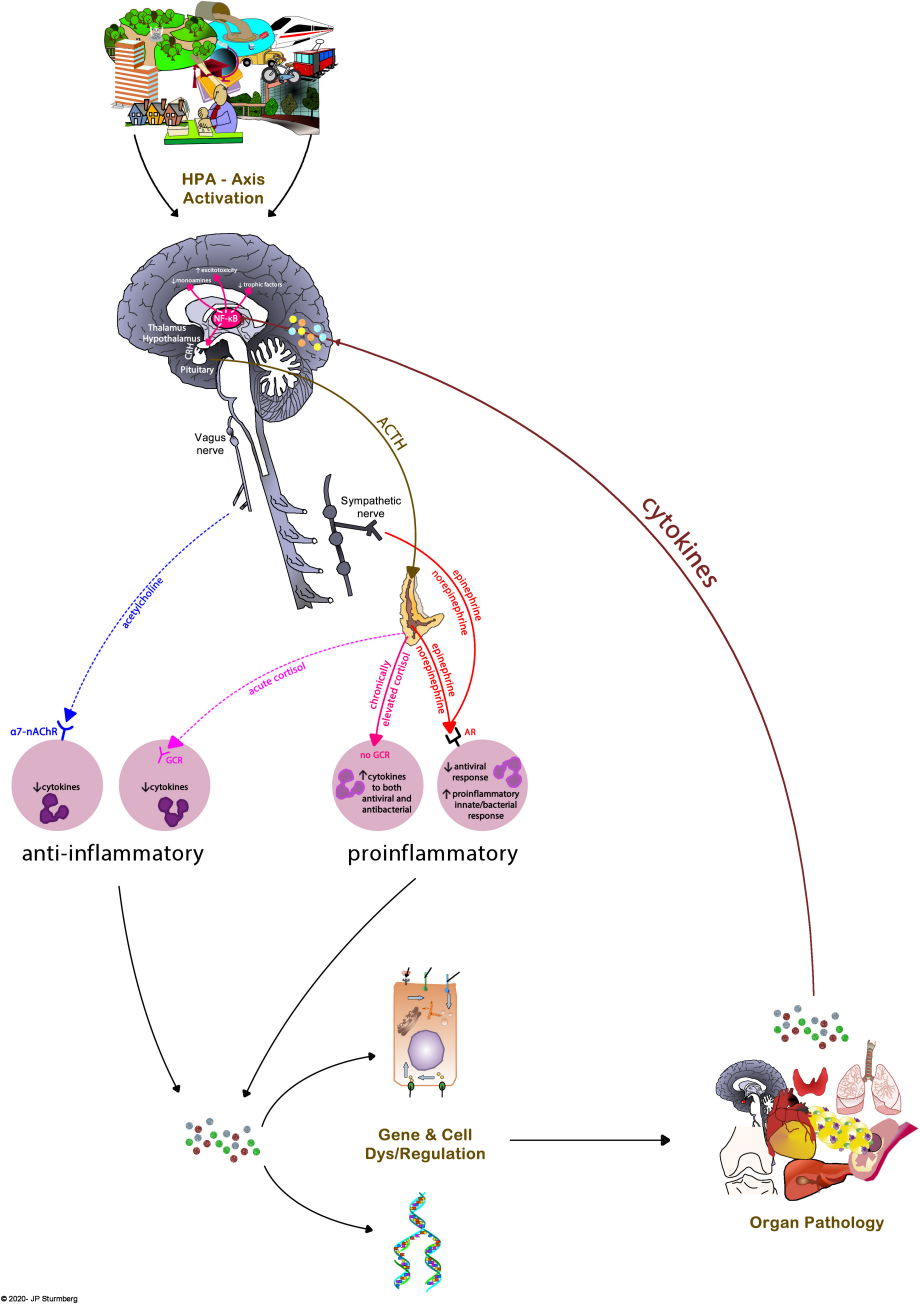
Overview of the interconnected nature of inflammatory dys/regulation, disease and health experience. The HPA-axis controls inflammatory regulation involving different pathways, all of which ultimately impact the balance between pro- and antiinflammatory cytokines. Cytokines are important mediators of gene and cell regulation, and thereby the emergence of organ pathology. In turn, organ pathology causes proinflammatory cytokines which impact health experience at the subjective, and HPA-axis activation at the objective levels. External environmental factors are a potent source of HPA-axis activation, the means by which “the environment gets under your skin.”

And finally, chronic overactivation of the HPA-axis triggered by external life events or circumstances (*stressors*) also causes chronic immune dysregulation and a pro-inflammatory state resulting in poorer health as exemplified in people from socioeconomically deprived communities (52, 53) (Figure 8).

Importantly, while we understand HPA axis *regulation* and while we have a fairly detailed understanding of its *regulatory pathways*, it remains yet unclear how the underlying various low-level physiological networks work in detail, and how they interact with each other (54). However, while these “microscopic” physiological details are missing, they are not always necessary to appreciate their observable “macroscopic” consequences (36).

#### Top-Down Causation in Complex Adaptive Systems

As indicated the higher-level external environment has major impacts on the intermediate level—physiological—function arising from the lower-level—biological—properties of our genetic/genomic blueprint.

It is the interactions between many different *modular hierarchical structures* that leads to the complexities of life in general and health at the personal level in particular (40). As Ellis explained: “*The basic principle is that when you have a complex task to perform, you break it up into subtasks that are each simpler than the overall project, requiring less data and less computing power, and assign these tasks to specific modules. Each module is again split up into submodules until you reach a base level where the necessary tasks are simple operations that can be carried out by simple mechanisms. This is the level where the real work is done, each of these components feeding its results into the next higher level components until the desired result emerges at the appropriate higher level. The modules at each level will interact with each other in some way. The result is a highly structured hierarchy of interacting entities*” (26).

A highly complex systems, like an organism, organ or cell, requires top-down causation (40). The necessary information to build such a highly complex system “*cannot be derived in a bottom-up way, because it implicitly embodies information about environmental niches. It would be different in a different environment. Hence, higher level conditions influence what happens at the lower levels, even if the lower levels do the work*” (26).

**Figure 9:**
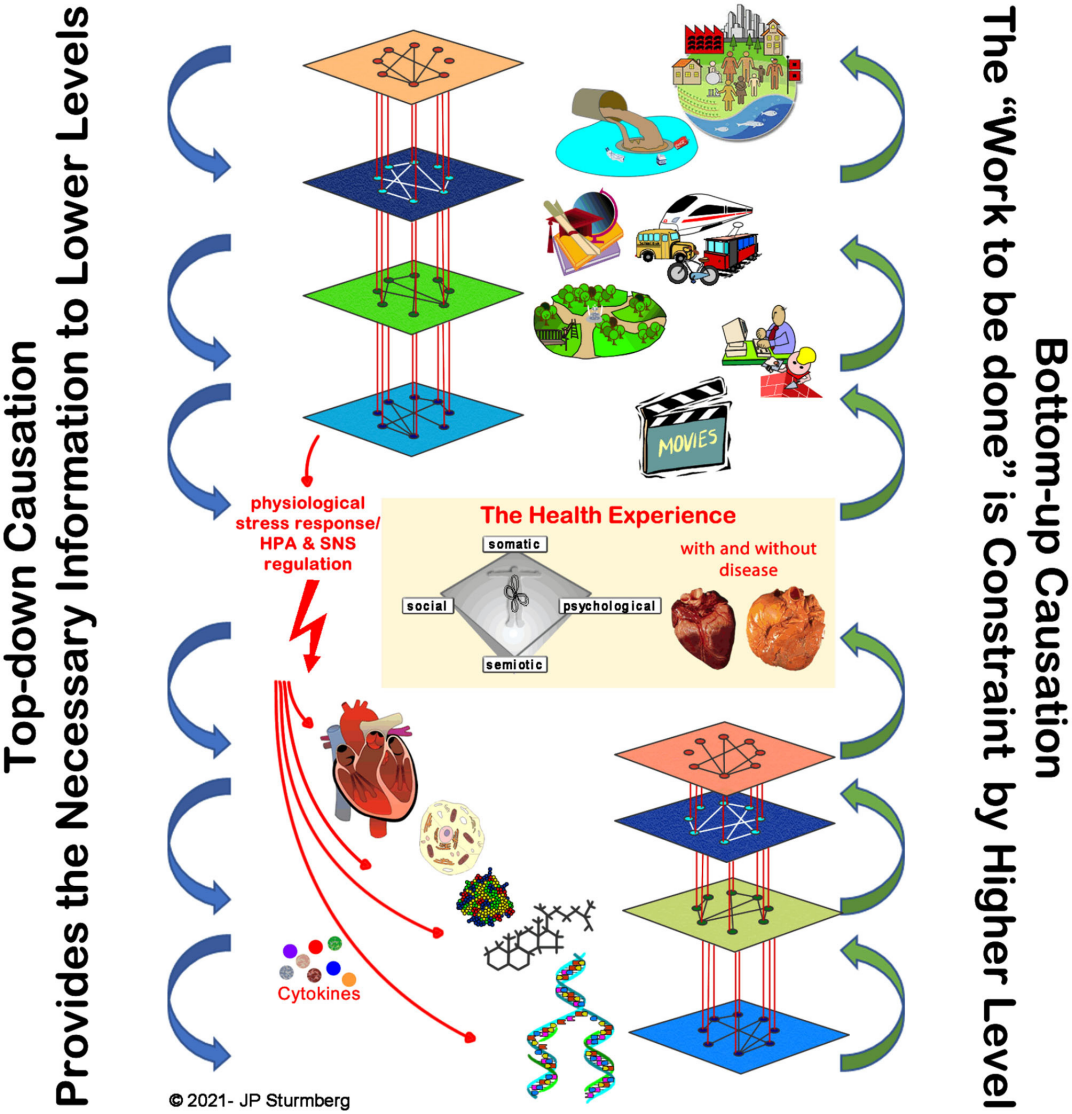
Top-down causation in health. Top-down causation is “necessary” for the function of complex adaptive systems. The higher-level external environment provides the necessary information to the lower levels that constrain their emergent possibilities, and thereby all them to “do the work” required to maintain life and health. The latter is particularly evident from network physiology research (36, 54, 55). For completeness the figure integrates the network relationships between the layers, as well as the main HPA-axis regulatory pathways.

In other words, while genes provide the information to create necessary biological building blocks, this is not sufficient to create or maintain life and health. This needs higher-level information to instruct lower levels to do the required work at a particular point in time in that particular context. Life and health require such *modular hierarchical system structures* to provide the adaptive dynamics to maintain both life and health (40) (Figure 9).

## Putting it all Together—The Emergence of a New Paradigm. Non-linear Complex-Adaptive Dynamics Result in Health, Dis-ease and Disease

The discourse so far has outlined that pattern formation can be explained mathematically through often rather simple equations. While health professionals are trained to recognize patterns as a means to diagnose diseases, they have as yet—very rarely—explored the underlying nature and dynamics that leads to these patterns. Furthermore, this discourse has elucidated core features that explain how people experience health as a personal state as much in the presence as absence of identifiable diseases. Basic sciences have shown, firstly, that physiological variables are non-linearly distributed (56), and secondly, that the non-linear networked dynamics across the macro- to nano-levels, largely controlled by the inflammatory cascade, can lead to the described different health states (see examples above).

Likewise, we find patterns in health care delivery. Epidemiological studies have repeatedly shown the pattern of health, dis-ease and disease distribution in the community follows a Pareto (80/20 split) pattern−80% of the community is healthy or healthy enough not to require health care, of the remaining 20%, 80% (i.e., 16% of the community) solely require primary, of the remaining 20%, 80% (i.e., 3.2% of the community) need secondary, and the remaining 20% (i.e., 0.8% of the community) tertiary health care services (57, 58).

Looking further 80% of patient consultations end with no clinical diagnosis; of those with a diagnosis 80% have 20% of all diagnosis (the common ones), whereas 80% of all diagnosis (the rare ones) occur in 20% of all patients (59). Clinical reasoning shows the same pattern, 80% of patients who did not get into a home (instead of a hospital-based) dialysis programme had limited patient education and lack of communication skills (60). Hospital service utilization also showed a Pareto distribution pattern—slightly more than 20% of emergency department presentations were attributable to adverse medication events, and 80% of these events were caused by 20% of all drugs prescribed by community-based physicians (61). And lastly, the 10 most common causes of death affect 80% of all mortals, whereas all other (rarer or rare) causes occur in the remaining 20% (62).

### New Paradigm Questions

The frame of health as a *dynamic complex-adaptive state* defines a *new paradigm*. Non-linear dynamics throughout a hierarchically layered complex-adaptive system explains the familiar pattern formations of health, dis-ease and disease. These understandings have far reaching consequences for health care (63).

The new frame puts to the forefront the question: *How are all of the patient's features across the macro- to nano-level connected and interacting to result in the presenting health state?*

Given that the HPA-axis dysregulation has emerged as the main regulator/integrator of the physiological networks, a closely related—macro-level focused question—ought to be: *What in this patient's life are the key triggers of HPA-axis dysregulation resulting in dis-ease and disease?*

The final—therapeutic—key question then has to be: *What are the consequences of dis-ease and disease on the person-as-a-whole, and how can we modulate any system features in such a way that they most likely tip the patient from a dis-ease to a health state?*

The *new paradigm* seeks to understand and manage the *interconnected and interdependent features of health* across the multiple networked layers of the person-as-a-whole, and supersedes the old paradigm focus on “dissecting disease/s” and treating each of these “in isolation”.

### The Challenges to Translate These Insights Into Clinical Practice

Scientific discourse and discoveries on their own are not sufficient to change policies and practices. As a health science community, we need to find translational answers to pragmatic concerns including amongst others:

- How do we ensure that health professionals broaden their approaches to patient care that enables them to explore *the whole* of their patient's dis-ease and disease presentations?
- How do we ensure that society at large understands the interconnected nature of health between their external environment perpetuating physiological dysfunction that determines health experiences and leads to disease development?
- How can we influence the policy settings that undermine health in general, and the health of deprived communities in particular?
- How do we create a care environment that enables whole-of-system care delivery?
- How do we monitor and adapt care, funding and policy processes in light of our achievements?

### Better Health Care and Better Health Outcomes Are in Our Grasp

The paper outlined the key building blocks for change that honors the Hippocratic oath to deliver health care that holistically embraces the somatic, psychological, social and cognitive-semiotic needs inherent in the person's health experience. We can (easily) do that, but it inevitably will entail to rethink and reorganize health service delivery. In particular, we need to recognize that, as a prerequisite to building healing relationships (64), a reorganized health service focused on whole-of-system care will need to allocate sufficient time and resources to care delivery (65) as well as upskilling its providers with greater system oriented communication skills (66). This seems to be an anathema to the still prevailing neo-liberal doctrine of the medical-industrial complex.

## Data Availability Statement

The original contributions presented in the study are included in the article/supplementary material, further inquiries can be directed to the corresponding author/s.

## Author Contributions

The author confirms being the sole contributor of this work and has approved it for publication.

## Conflict of Interest

The author declares that the research was conducted in the absence of any commercial or financial relationships that could be construed as a potential conflict of interest.
